# Sutures benefit not only wounds

**DOI:** 10.1192/bjb.2019.9

**Published:** 2019-04

**Authors:** Ann Maria Albert, Karel Wildschut, Marinos Chatzopoulos, Johanna Selway

**Affiliations:** GPST1, Oxford GPVTS, email: annmaria.albert@nhs.net; ST6 in Old Age Psychiatry and CAT practitioner trainee, Cambridge and Peterborough FT; Consultant Psychiatrist, Metropolitan General Hospital, Athens, Greece; ST3 ACCS Emergency Medicine, East of England.

We compliment Buick and colleagues^1^ for their innovative venture. We want to stress that the benefits of in-house management of minor wounds are far more than financial.

## Reputation

Many foundation year, general practitioner and psychiatry core trainees go to psychiatry after doing their surgery and Accident & Emergency (A&E) posts, where they would have managed many wounds with glue, staples and sutures. However, while in psychiatry, they are ‘duty-bound’ to refer patients with self-inflicted superficial wounds to the A&E. This arrangement would make the A&E staff and others wonder why junior doctors relinquish this role when moving to psychiatry, especially if a few months earlier this had been part of their day-to-day role.

## Self-esteem

When junior doctors who are skilled in the management of superficial wounds are forced by mental health trusts to refer patients with superficial wounds to the A&E department, many junior doctors will wonder why they are no longer being seen as suitable and competent enough to carry out this procedure.

## Costs to other patients

Most psychiatric in-patient wards are understaffed. Buick and colleagues estimated the nursing costs under the presumption that only one nurse would go to A&E with the patient. Often, two or more nurses would have to accompany the patient to manage risks, including that of absconding. Nurses taking patients to the A&E for management of superficial wounds would result in less staff on the ward and less nursing care for those patients who do not self-harm.

## Stigma

Taking every patient with superficial wounds to A&E would perpetuate the stigma about mental illness, mental health patients, services and staff.

## Perpetuation of self-harm behaviour

At least for some patients who self-harm, being the centre of attention, and being taken to the A&E by ambulance and accompanied by one or more nurses is likely to perpetuate their self-harm behaviour.

The psychological dimensions of self-harm behaviour can be complicated. The cognitive analytic model offers an understanding of how relationship roles might play out in self-harm, whether self-to-self or self-to-other.

For some patients, self-harm can elicit unhelpful, reinforcing reciprocal roles and procedures, which may seem obvious if judged at face value only. Continuing to enact these roles may delay the adoption of more sustainable and healthy ways of dealing with distress. Being accompanied to A&E by caring professionals and receiving restorative care and attention there – while appropriate and wholesomely good – might feel rewarding. It has the potential to evoke a rescuer-to-rescued reciprocal role, or perhaps a caring-to-cared for role, but at substantial personal cost.^2^^–^^4^

For many patients, the same situation can indeed feel disempowering and punishing when they are not given a choice in how we care for them in the aftermath of self-harm. The National Institute for Health and Care Excellence emphasises the need for people to be fully involved with decision-making about their treatment and care.^5^

In the same context, it is possible for the patient to experience a more direct reward. If the social reinforcement of self-harm is rewarding enough, the individual may inflict more severe pain in order to continue receiving that positive reinforcement from their environment.^6^

## Mental health trusts’ view

Often, mental health trusts object to junior doctors managing superficial wounds based on unsubstantiated arguments, e.g.:

• how do we prove that the junior doctor is competent?
• what will happen if patients complain about scars?
• how do we ensure that the equipment is of the required standard?
• If they do so, both the trainee and the consultant need to be very clear that they are doing this outside any Trust governance structures and under their own assessment of the risks and consequences. There are no supporting Trust policies or guidance for this. It is a minefield for litigation.

## The way forward

It would be helpful for the College to take a stand and advise the mental health trusts about the minimum services that need to be provided, to ensure that junior doctors have the necessary skills and enable them to provide the services that they are competent to deliver.

## References

[1] Buick TA, Hamilton D, Weatherdon G, O'Shea CI, McAlpine G. Evaluating the effects of a peer-led suturing and wound management workshop for doctors working in a psychiatric hospital. BJPsych Bull 2008; 42; 211–6. 10.1192/bjb.2018.41 PMC6189990 30345069

[2] Denman C. Cognitive–analytic therapy. Adv Psychiatr Treat 2001; 7: 243–52.

[3] Ryle A, Kerr IB. Introducing Cognitive Analytic Therapy: Principles and Practice. Wiley-Blackwell, 2002.

[4] Chapman AL, Gratz KL, Brown MZ. Solving the puzzle of deliberate self-harm: the experiential avoidance model. Behav Res Ther 2006; 44: 371–94. 16446150 10.1016/j.brat.2005.03.005

[5] National Institute for Health and Care Excellence. Self-Harm in Over 8s: Long-Term Management. Clinical Guidance CG133. NICE, 2011 (https://www.nice.org.uk/guidance/cg133). 31891461

[6] Lloyd-Richardson E, Perrine N, Dierker L, Kelley M. Characteristics and functions of non-suicidal self-injury in a community sample of adolescents. Psychol Med 2007; 37: 1183–92. 17349105 10.1017/S003329170700027X PMC2538378

